# Effect of Pulsed Electric Field on the Chicken Meat Quality and Taste-Related Amino Acid Stability: Flavor Simulation

**DOI:** 10.3390/foods12040710

**Published:** 2023-02-06

**Authors:** Ume Roobab, Xin-An Zeng, Waqar Ahmed, Ghulam Muhammad Madni, Muhammad Faisal Manzoor, Rana Muhammad Aadil

**Affiliations:** 1 School of Food Science and Engineering, South China University of Technology, Guangzhou 510641, China; 2 Overseas Expertise Introduction Center for Discipline Innovation of Food Nutrition and Human Health (111 Center), Guangzhou 510640, China; 3 Guangdong Provincial Key Laboratory of Intelligent Food Manufacturing, Foshan University, Foshan 528225, China; 4 National Institute of Food Science and Technology, University of Agriculture, Faisalabad 38000, Pakistan

**Keywords:** PEF, amino acid, chicken, flavor, quality, intensity, meat color

## Abstract

Meat contains several amino acids related to taste, which have a significant impact on the overall acceptability of consumers. A number of volatile compounds have been studied in relation to meat flavor, but amino acids have not been fully explored in relation to the taste of raw or cooked meat. It would be interesting to find any changes in physicochemical characteristics, especially the level of taste-active compounds and flavor content during non-thermal processing such as pulsed electric fields (PEF), for commercial reasons. The effect of PEF at low intensity (LPEF; 1 kV/cm) and comparatively high intensity (HPEF; 3 kV/cm) with different pulse numbers (25, 50, and 100) was investigated on the physicochemical characteristics of chicken breast, including the free amino acid content (related to umami, sweet, bitter, or fresh pleasant taste). PEF is regarded as a “nonthermal” technology; however, HPEF induces moderate temperature rises as it increases with the treatment intensity (i.e., electric field strength and pulse number). The pH, shear force, and cook loss (%) of the LPEF and untreated samples were not affected by the treatments, but the shear force of the LPEF and untreated samples was lower than that of HPEF groups that showed PEF-induced slight structural modifications resulting in a more porous cell. In the case of color parameters, the lightness of meat (L*) was significantly higher with treatment intensity, whereas both a* and b* were unaffected by the PEF treatments. Moreover, PEF treatment significantly (*p* < 0.05) affected umami-related free amino acids (FAAs; glutamic acid and aspartic acid) and leucine and valine, which are precursors of flavor compounds. However, PEF decreases the level of bitter taste contributing FAAs such as lysine and tyrosine, which may prevent the formation of fermented flavors. In conclusion, both PEF treatments (LPEF and HPEF) did not adversely impact the physicochemical quality of chicken breast.

## 1. Introduction

Pulsed electric field (PEF) is still an emerging nonthermal technology in preserving food and enhancing the quality characteristics of food [1]. Food quality is associated with an equally important trend in the food industry: consumers’ increased interest in goods that bring health benefits, i.e., increase immunity or provide essential nutrients. Nonthermal technologies process foods with more “fresh-like” flavor than those produced by conventional thermal processing due to some physicochemical modifications [2]. Unlike conventional methods, PEF allows the product to maintain its physical and chemical properties. For instance, PEF-treated samples (0.8–1.1 kV/cm, pulse width of 20 μs, frequency of 50 Hz) improved meat tenderness and color and maintained the physicochemical and sensory properties of beef muscles till the 7th day of storage [3]. Similarly, PEF treatment of frozen-thawed beef samples (FP) resulted in tenderization, as shown by a 20.13% (*p* < 0.01) decrease in the average maximum shear force compared to the frozen-thawed control [4]. In the last decade, PEF technology has been applied to a variety of prototype food products [5], for quality enchantment [6], pasteurization [7], enzymes inactivation [8,9], fresh-cut processing [10], shelf life and nutritional value enhancement [11], valuable compound extraction [12,13,14], food waste valorization [15], and so on. The use of this technology is still scant for meat processing at a commercial scale; however, it has been explored as a sodium-reducing strategy for meat products [16], nonthermal decontamination [17], improving the digestibility of cooked meat [18], freeze-thaw quality, meat ageing [19], meat drying [20], and so on.

The effect of PEF treatments on amino acid content has been reported in beef [3], lamb [21], and seafood [22]. To our knowledge, no prior studies have examined the effects of PEF processing on amino acid content and release in chicken meat. Although PEF is currently under consideration for exploring its impact on the sensory properties of meat, including flavor enhancement strategies, the available published research relates to lean muscles. Therefore, this is a challenge for commercial systems to become a reality. Moreover, different PEF treatments would be needed for different muscles and cuts in different species for optimum results [23]. Different cuts and muscles for different species require optimum treatment parameters. The majority of published research has focused on using PEF on homogeneous meat samples or minor cuts rather than whole commercial cuts. In this article, some technological features of PEF-treated whole-cut chicken breast are described that directly impact meat quality, such as pH value, temperature, color, taste-related amino acids, cooking loss, and textural quality as affected by PEF treatments.

## 2. Material and Methods

### 2.1. Sample Collection

Commercial chicken breasts (*Pectoralis major*) were purchased from a supermarket (Guangzhou, China) from the same batch of broilers, similar in weight, sex, and age, and chilled at 4 °C ± 1. Samples thus obtained were weighed and separately vacuum-packed in low-density polyethylene bags.

### 2.2. Determinations of PEF Power and Intensity

**Figure 1:**
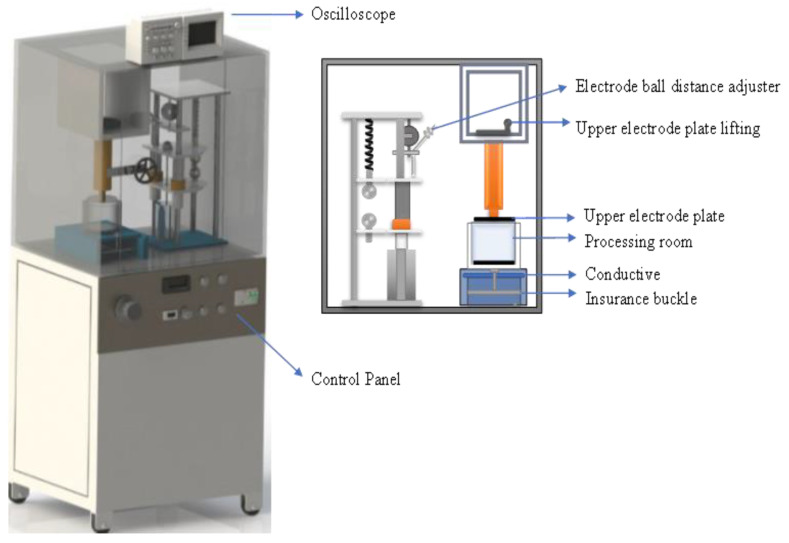
Schematic diagram illustrating the equipment used in batch systems with pulsed electric fields.

Treatment parameters were selected based on reports stating that PEF treatments of 0.6 kV/cm or higher electric field strength induce electroporation in animal tissues [24]. PEF treatments were applied through laboratory-scale PEF equipment (PEF-EX-1900, Guangzhou Xinan Food Technology Co., Ltd., Guangzhou, China), delivering a high voltage of 20 kV/cm (Figure 1). The pulse generator offers exponentially decaying monopolar pulses (Table 1).

In this experiment, whole chicken breasts (weighing approximately 170–180 g) were processed in a chamber (100 mL) consisting of two parallel stainless-steel electrode plates (3 mm thick) with an adjustable gap (0–10 cm). An oscilloscope (Tektronix TBS1102B) was used to monitor output voltage and pulses. Randomly selected samples were divided into experimental groups according to their PEF treatments (Table 2). It was ensured that the electric field delivered to the muscle fibers between the electrodes was perpendicular to the chicken breast samples. To facilitate electroporation, samples were dipped in tap water, and PEF treatment was applied at room temperature. There was no treatment applied to the control samples.

### 2.3. pH and Temperature Measurements

A calibrated Hanna pH meter and electrode (Model HI 98150) were used at ambient temperature to measure the pH of the PEF-treated samples immediately after treatment. The temperature was measured from the center of each sample using a combination puncture pH electrode immediately before and after PEF treatment. The results are reported as the change in sample temperature (the temperature after treatment minus the temperature before treatment).

### 2.4. Colorimetric Analysis

Color values L*, a*, and b* (D65 illuminate and 2° observer) were measured according to the method described by Baldi [1]. The samples were covered with oxygen-permeable polyvinylchloride film. Color measurements of samples were obtained before and after the treatments using a standardized chromameter (CR-400, Konica, Japan). Before the measurement, the instrument was calibrated with a standard white tile. L* values measure darkness to lightness (larger values indicate a lighter color), while a* values measure redness (positive values indicate a more red color, and negative values indicate a more green). However, b* values measure yellowness (positive values indicate the degree of yellow and negative values indicate degree of blue). For each sample, at least five measurements were performed at different positions.

### 2.5. Identification and Quantification of FAAs

The FAAs were analyzed according to the method described by Shimamura [25]. The samples (10 g) were homogenized in 40 mL of 2% (*w*/*v*) sulfosalicylic acid solution, and centrifuged (JW-3021 HR, Anhui Jiaven Equipment’s Industry Co., Ltd., China) at 3000× *g* for 10 min, subsequently centrifuged (inner layer) at 10,000× *g* for 10 min. The supernatant was filtered (0.45 μm) and amino acids were detected using a fully automated amino acid analyzer (Hitachi L-8800, Hitachi Co., Ltd., Tokyo, Japan). All the outcomes were expressed in ng/20 µL.

### 2.6. Determination of Shear Force and Cooking Loss

The shear force (Newtons) and cooking loss (%) measurements were carried out as described by Khan [26] with some modifications. Chicken breast samples were cut into 3.0 × 2.0 × 0.4 cm (L × W × H) pieces. The shear force values of raw chicken breast samples were measured using a Warner-Bratzler V-shaped shear blade (5.0 mm/s test speed) TA. XT-plus Texture Analyzer (Serial No. 12835, Stable Micro Systems, Surrey, UK). During the texture analysis of cooked samples, each bag of samples was submerged in a water bath and cooked at 80 °C until the internal temperature reached 75 °C, measured with a Fluke thermometer. The samples were immediately cooled in ice before being blotted dry with paper towels, weighed, and cut perpendicularly. The results were recorded for three replicates of each treatment and expressed as the samples’ shear force (N). For cooking loss (%), the difference in weight before and after cooking was calculated by the formula below:

$$\mathrm{Cooking}\;\mathrm{loss}\;\%=100\times \frac{\mathrm{weight}\;\mathrm{before}\;\mathrm{cooking}\;-\;\mathrm{weight}\;\mathrm{after}\;\mathrm{cooking}}{\mathrm{weight}\;\mathrm{of}\;\mathrm{the}\;\mathrm{sample}\;\mathrm{before}\;\mathrm{cooking}}$$

### 2.7. Statistical Analysis

All experiments were repeated three times, except for FAA, which was the average of two samples. The general linear model was used in Minitab (version 16.2.4), and the difference among individual group means was evaluated by the Tukey test [27]. The results of all analyses were shown as mean values and standard deviations (SDs), and *p* < 0.05 values were regarded as statistically significant.

## 3. Results and Discussion

### 3.1. Effect of PEF Treatments on pH and Temperature

According to previous research, PEF treatments have a mixed effect on meat pH levels. Regardless of the applied strength of the electric field and the number of pulses, PEF treatments were ineffective for raising meat pH, as seen in Table 2. Baldi [1] found that PEF treatments (0.60–1.20 kV/cm, 150–600 pulse numbers) had no significant impact on the pH values of chicken breast. However, these results contradict Khan [26], as they observed a significantly (*p* < 0.05) decreased pH (by 0.16 units) of chicken breast meat after HPEF (10 kV/cm) treatments. However, in the case of beef meat, these results are entirely contrary to those observed by Faridnia [28]. When beef samples were treated with LPEF at 1.7 kV/cm, pH decreased significantly. The authors conclude that increased broken myofibrils along Z-lines resulted in a more porous structure. However, it was agreed that low-pH muscles have less shear strength [24]. Earlier work by Faridnia [29] and Bekhit [24] suggested that the pH of LPEF-treated beef (0.2–0.6 kV/cm) and HPEF-treated beef (10 kV/cm) was not affected by PEF parameters.

**Figure 2:**
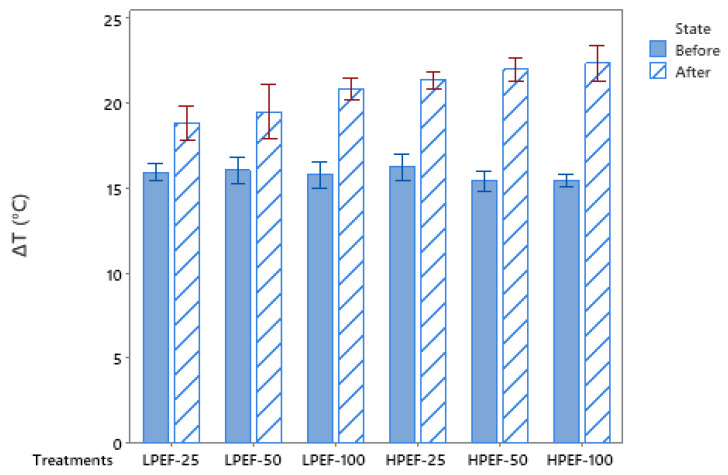
Effect of different pulsed electric field treatments on temperature change ΔT (°C) of chicken meat samples.

Our study showed significant temperature fluctuations due to PEF treatments **(**Figure 2). This was confirmed by Khan [26], who found that HPEF (10 kV, 200 Hz, and 20 μs)-treated chicken samples experienced a more pronounced (*p* < 0.001) temperature shift after treatment (ΔT = 17.1 °C) compared with LPEF (2.5 kV, 200 Hz, and 20 μs)-treated samples (ΔT = 3.1 °C). According to Arroyo [30], the temperature rose by 7.7 °C at 300 pulses and 14.5 °C at 600 pulses (20 s) with a 1.4 kV/cm PEF treatment when applied to beef samples. Faridnia [28] achieved similar results by using PEF treatments (at 1.7 kV/cm) on beef, which significantly increased the temperature (26.5 °C). Further support is given by Bekhit [24] that using HPEF (10 kV/cm) treatment induces protein denaturation without adversely affecting beef quality. However, for the present study, it can be hypothesized that the increase in temperature (<30 °C) was not severe enough to cause denaturation. It has been reported that temperatures above 40 °C can increase the lethality of PEF, despite the fact that the temperatures in this study reached below the threshold for microbe mortality (50 °C).

### 3.2. Changes in Color

The main factor affecting customers’ buying preferences is meat color, which has only been examined in a few studies (discussed later in this section). U.S. consumers prefer lighter-colored poultry meats (especially breast meat) to darker-colored ones, according to a recent study [31]. Additionally, Sow [32] determined the sensory characteristics and consumer preferences for chicken in Guinea, showing a high preference for yellow-colored live village chicken meat. Alternatively, Chinese consumers prefer dark meat and chicken wings and feet, while breast meat is significantly less expensive [33]. However, myoglobin’s redox state could be altered during PEF treatments due to temperature increases, which might affect PEF’s impact on meat color [34]. Nevertheless, increasing pulse counts combined with high-intensity treatments can promote myoglobin oxidation and meat discoloration due to increased sample temperature [35]. Although comparatively low-intensity treatments (<5 kV/cm) were applied in this study, PEF slightly affected meat lightness (L*), whereas redness and yellowness (a* and b*) were unaffected (Table 2**)**. Recently, Baldi [1] achieved similar results using LPEF treatments at 0.60–1.20 kV/cm electric field strength and 150–600 pulses [1]. However, the findings contrast with the data reported by Khan [26], who observed a higher a* value at LPEF (2.5 kV/cm) than control samples, which did not affect L* values, but there may be many reasons for this. It would be reasonable to expect that color changes were not linked to the temperature fluctuations in samples during the treatment but rather to the possible redistribution of water within the cells following PEF [1]. Certainly, PEF might have caused the transport of water inside cellular compartments, altering tissue refractive properties [36]. However, as discussed in the literature, milder PEF intensity did not change the color parameters of the beef and turkey samples [30,37]. Interestingly, in this study, meat color differences in PEF-treated samples were negligible and probably not noticeable to the human eye.

### 3.3. Effect of PEF Treatments on FAAs

Amino acids are the building blocks of proteins, and they determine the quality of meat. Additionally, certain amino acids (due to their composition and content in meat) play an important role in establishing the flavor properties of foods, as some impart unique flavor characteristics while others act as precursors to odors and other flavor compounds [38]. Several amino acids have distinct tastes; for example, arginine, isoleucine, leucine, valine, phenylalanine, methionine, and histidine impart a bitter taste. In contrast, glutamic acid and aspartic acid possess a pleasant fresh taste, while glycine, alanine, and serine impart a sweet taste [39]. 17 FAAs were recorded and compared between the groups in this study. As seen in Table 3, the HPEF treatments showed more total FAA content compared to the LPEF group and control group (*p* < 0.05). However, PEF treatments had no significant (*p* > 0.05) effect on alanine and proline content. Furthermore, the umami-related FAAs (glutamic acid and aspartic acid) were remarkably intensified under different PEF treatments compared to the control samples. As a result of these FAAs, the flavor and aroma of products are likely to have an improved sensory quality [40]. However, aspartic acid content was higher in HPEF-100 compared to LPEF-25 and control samples. While LPEF-25 showed better performance in intensifying glutamic acid (1219.7). The low PEF treatment and comparatively high PEF treatment groups contained similar amounts of sweet FAAs (i.e., alanine). Furthermore, the samples treated with LPEF-25 showed the lowest concentrations of aspartic acid, threonine, alanine, leucine, isoleucine, tyrosine, phenylalanine, valine, methionine, lysine, arginine, and proline as compared to other treatments. While the samples treated with HPEF-100 showed the highest concentrations of threonine, serine, alanine, and methionine compared to other treatments. It is plausible to hypothesize that increasing the treatment intensity (electric field and pulse number) impacted protein structure in several ways, such as unfolding and denaturation [41,42] and thus influenced the release of FAAs. Other researchers also demonstrated the increase of FAAs induced by the PEF treatments in tea [43], juice [44], dates [45], and meat [46]. Another explanation is that electric fields might damage a protein’s double-layer structure and destroy its n-potential, resulting in the degradation of small proteins or peptides into FAAs. According to this study, it is possible that some soluble proteins were hydrolyzed or embedded FAAs were released, causing an increase in total FAAs and individual FAAs. Further investigation can be undertaken to explore the mechanism of the increased FAAs in PEF-treated meat samples.

In addition, leucine and valine (important precursors of flavor compounds) were significantly higher in HPEF-25 (*p* < 0.05) as compared to the LPEF group. The is primarily caused by the conversion of aminotransferases into α-keto acids, which are then metabolized to aroma compounds such as aldehydes (impart malt-flavor), alcohols (impart fruit-flavor), and acids (impart ripe flavor) [47,48]. Despite FAAs being critical precursors of flavor development, high concentrations of some FAAs (such as lysine and tyrosine) may promote biogenic amines with fecal and putrid off-flavors, which can negatively affect meat flavor by enhancing decarboxylase activity [49]. However, the PEF treatments may prevent the formation of fermented flavors. For instance, increasing the pulse number (from 25 to 100) increased the lysine (from 334.6 to 505.3 ng/20 µL) and tyrosine (from 274.7 to 339.03 ng/20 µL) in LPEF groups. However, increasing the electric field decreased the lysine (from 417.8 to 374.9 ng/20 µL), and tyrosine (from 353.2 to 348.09 ng/20 µL) as in the case of HPEF groups. Similarly, most of the FAAs contributing to bitter taste, especially phenylalanine, significantly decreased (from 309.5 to 241.4 ng/20 µL) while increasing the pulse number (from 25 to 100) in HPEF groups. In conclusion, the PEF treatments significantly increased taste-related amino acids in the breast muscles, including umami and sweet taste FAAs. In contrast, the bitter taste phenylalanine and cysteine were reduced (*p* < 0.05) in PEF-samples compared to the control. It could be assumed that PEF treatments induced the denaturation of protein molecules and released FAAs, as previously stated in the literature [50]. Moreover, the concentration and composition of FAAs in various types of meat also depend on other before- and after-slaughter factors, such as animal type/breed, diets, animal handling, slaughtering, and storage conditions. However, there are still limited advanced systematic approaches to assess these taste-related compounds. 

### 3.4. Effect of PEF Treatments on Texture (Shear Force) and Cooking Loss (%)

**Figure 3:**
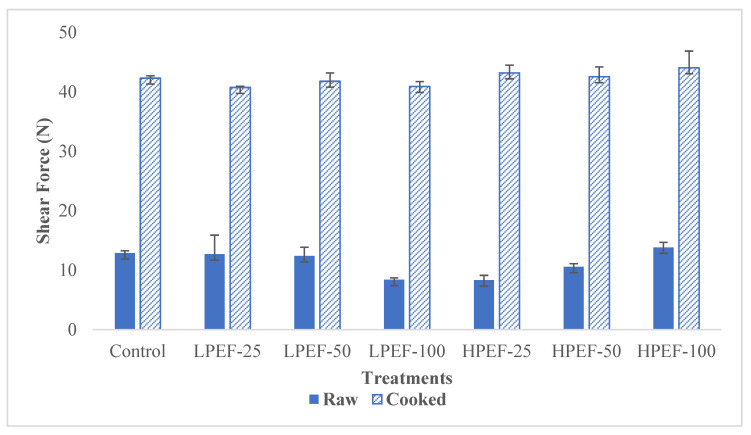
Effect of different pulsed electric field treatments on the shear force (N) of chicken meat samples compared to the untreated control (control).

**Figure 4:**
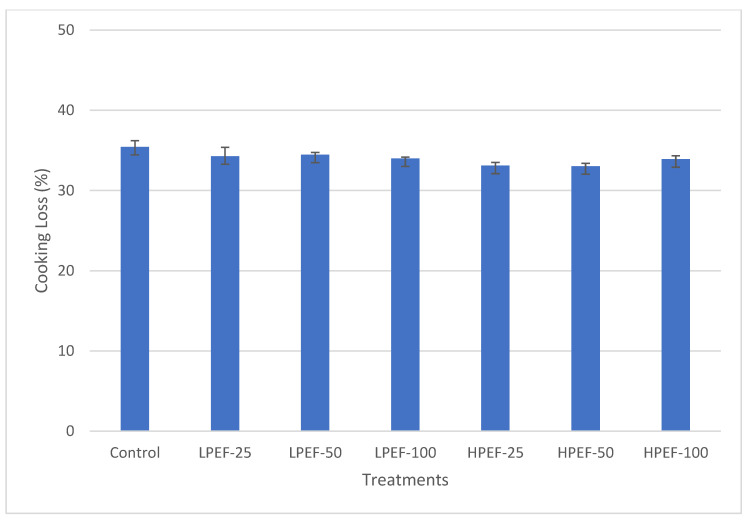
Predicted means for the cooking loss (%) of treated chicken breasts.

In this study, the shear force and cooking loss of low- and relatively high-intensity PEF-treated samples were compared with those of untreated samples. Instrumental texture measurements indicated that the shear force (N) of both raw and cooked chicken breasts (Figure 3) and cook loss (%) were unaffected by treatment (*p* > 0.05) (Figure 4). Several studies found a nonsignificant impact of PEF (0.2–10 kV/cm) on the shear force of beef (*Longissimus thoracis*) muscles [26,29,30] and cooking loss [28,29]; however, the shear force of LPEF and an untreated sample was lower than HPEF samples. O’Dowd [51] reported similar observations in their experiments on beef muscle (*Semitendinosus*), as the texture was unaffected by the PEF treatments, i.e., 2.8 kV/cm for pulse number (152–300). According to the authors, the LPEF treatments may not have been intense enough to physically disrupt fibers to a point where they could affect tenderization. The authors explained that the LPEF treatment enhanced the permeabilization of animal tissues, which could enhance proteolysis [52,53]. Similarly, Arroyo [37] found no effect on the shear force at 7.5, 10, and 12.5 kV/cm (fresh meat) and 14, 20, and 25 kV/cm (frozen meat) of turkey breast meat. In contrast, PEF treatments (1.7 kV/cm, 50 Hz, 20 µs) significantly reduced the shear force of beef outside flat (*Biceps femoris*) samples; however, cooking loss was unaffected [28]. The reduction in shear force might be caused by the rupture of myofibrils along the Z-lines, resulting in increased porous muscle structure, according to Bekhit [52]. However, cooking effects appeared on the meat edges at 10 kV/cm PEF intensity. While the reduced shear force might be due to the increased number of ruptured myofibrils along the Z-lines, making muscle structure more porous [28]. Similarly, shear force values in beef samples were reduced by 22.5% for *Triceps brachii* muscles after PEF (3.5 kV/cm, 100 pulses) and by 19.5% for the *longissimus lumborum* and *semimembranosus* muscles (after PEF at 0.27–0.56 kV/cm [52,54]. It is hypothesized that PEF induces cell permeabilization, which in turn increases endogenous enzymes responsible for meat’s textural changes. Although there is a pervasive literature on the impact of PEF treatments on beef, lamb, and turkey samples [23], no previous study has used this technique to simultaneously investigate the impact of shear force on raw and cooked chicken breast samples. In our study, it is evident that the HPEF-treated and control groups exhibited mathematically higher shear force values than LPEF samples for raw and cooked meat.

On the other hand, Khan [26] investigated the effects of LPEF (2.5 kV/cm) and HPEF (10 kV/cm) on chicken breast quality at 1 and 4 days after PEF treatments. PEF-treated samples showed no significant (*p* ≥ 0.05) changes in cooking loss (%) as compared to control samples. The cooking loss % of LPEF and an untreated sample, on the other hand, was higher than that of HPEF samples. As a result of these studies, PEF does not appear to negatively affect meat juice loss during cooking. In spite of the lack of agreement in the literature about whether PEF treatments improve meat tenderness, there are several factors that influence the complex effect of PEF treatment on meat, including the intensity of the treatment, the processing chamber, the muscle type, and sample pretreatments.

## 4. Conclusions

Overall, our results showed that PEF treatments (applied in the experiments) improved the taste-related amino acids without affecting the textural properties or damaging proteins. Further investigation can be undertaken to better understand the mechanisms involved in enhancing flavor compounds after PEF treatments and flavor preservation during meat processing, cooking operations, and storage conditions. The PEF method has some potential technological impact on chicken meat that might be used in the future for commercial purposes. However, more research is required to establish the optimal inputs (such as electric field strength, frequency, processing time, number of pulses, and so on) for different muscles, cuts, and species.

## Figures and Tables

**Table 1:** Technical data of the PEF-EX-1900 batch system (Xi-nan Technology Company, China).

Parameters	Specifications
Input power supply voltage	220 V, 50 Hz
Adjustable discharge mode	Direct high-voltage rapid discharge.
Output maximum discharge voltage	0–20 kV/cm
Electrodes	Stainless steel, parallel plate, 200 × 200 mm
Electrode gap	0–10 cm, adjustable distance.
Treatment chamber material	Plexiglass
Output Power	≥500 W
Output frequency	0–1.5 Hz (the higher the voltage, the longer the charging time, and then the lower the frequency)
Processing chamber volume	Configuration: 100 mL, 400 mL, and 700 mL, three kinds of containers.
Pulse shape	Exponential decay pulses, monopolar
System capacity	1 μF

**Table 2:** Effect of different PEF treatments on pH and color content of chicken meat samples.

Treatment	Code	Electric Field Strength (kV/cm)	Pulse Number	pH	L*	a*	b*
Control	Control	–	–	5.98 ± 0.12	42.51 ± 0.36 ^ab^	1.58 ± 0.08	6.12 ± 0.71
Low-PEF	LPEF-25	1.5 ± 0.13	25	5.96 ± 0.01	45.17 ± 0.85 ^ab^	1.29 ± 0.17	6.75 ± 0.46
	LPEF-50	1.5 ± 0.17	50	5.93 ± 0.02	45.78 ± 0.94 ^a^	0.93 ± 0.24	5.78 ± 0.31
	LPEF-100	1.5 ± 0.12	100	5.95 ± 0.03	43.12 ± 1.04 ^ab^	1.22 ± 0.22	5.98 ± 0.6
High-PEF	HPEF-25	3.3 ± 0.15	25	5.91 ± 0.02	43.04 ± 0.56 ^ab^	1.17 ± 0.2	6.59 ± 0.51
	HPEF-50	3.3 ± 0.16	50	5.93 ± 0.03	42.26 ± 0.55 ^b^	1.38 ± 0.11	6.82 ± 0.41
	HPEF-100	3.3 ± 0.10	100	5.90 ± 0.03	43.38 ± 0.71 ^ab^	1.04 ± 0.18	6.19 ± 0.22

The values are the predicted means ± SD. Within each column, the means have different pulsed electric field treatments. Superscripts are significantly different at *p* < 0.05.

**Table 3:** Free amino acid contents of chicken breast influenced by different pulsed electric field treatments.

FAAs Type (ng/20 µL)	Control	LPEF-25	LPEF-50	LPEF-100	HPEF-25	HPEF-50	HPEF-100
***Umami taste FAAs***							
Aspartic acid	454.07 ± 4.73 ^ab^	434.97 ± 0.93 ^b^	552.2 ± 1.33 ^a^	513.8 ± 10.2 ^ab^	525.1 ± 28 ^ab^	436.1 ± 5.92 ^ab^	462.7 ± 45.7 ^ab^
Glutamic acid	875.5 ± 5.59 ^d^	1219.7 ± 2.87 ^a^	1130.4 ± 5.81 ^ab^	951.1 ± 19.3 ^cd^	1047.7 ± 57.6 ^bc^	945.4 ± 6.55 ^cd^	1031.3 ± 3.09 ^bc^
***Sweet taste FAAs***							
Threonine	443.2 ± 10.7 ^c^	379.6 ± 8.52 ^d^	478.7 ± 6.49 ^abc^	465.1 ± 14.4 ^bc^	504.7 ± 18 ^ab^	508.02 ± 1.97 ^ab^	531.4 ± 2.2 ^a^
Serine	468.6 ± 1.05 ^c^	491.3 ± 1.21 ^bc^	512.8 ± 3.05 ^b^	500 ± 11.8 ^b^	507.1 ± 4.41 ^b^	516.99 ± 1.58 ^b^	565.5 ± 4.2 ^a^
Glycine	400.2 ± 1.54 ^ab^	371.3 ± 0.63 ^ab^	414.9 ± 2.4 ^ab^	409.5 ± 8.26 ^ab^	439.8 ± 19 ^a^	358.7 ± 1.24 ^b^	425.3 ± 25.3 ^ab^
Alanine	717.7 ± 4.59 ^a^	577 ± 136 ^a^	732.4 ± 3.79 ^a^	726.8 ± 16.4 ^a^	838.6 ± 23.4 ^a^	762.8 ± 9.8 ^a^	848.8 ± 4.68 ^a^
***Bitter taste FAAs***							
Isoleucine	248.7 ± 2.16 ^ab^	229.7 ± 0.39 ^b^	288.8 ± 1.31 ^a^	274.1 ± 6.1 ^a^	291.6 ± 7.02 ^a^	254.9 ± 2.36 ^ab^	264.9 ± 18 ^ab^
Leucine	472.5 ± 3.21 ^cd^	443 ± 0.32 ^d^	544.7 ± 3.02 ^ab^	511 ± 11.5 ^bc^	559.5 ± 2.03 ^a^	523.6 ± 6.95 ^ab^	522.2 ± 14.2 ^ab^
Tyrosine	294.6 ± 3.26 ^b^	274.7 ± 0.18 ^b^	338.1 ± 0.9 ^a^	339.03 ± 9.06 ^a^	353.2 ± 5.16 ^a^	340.02 ± 0.45 ^a^	348.09 ± 4.35 ^a^
Phenylalanine	967.8 ± 4.87 ^a^	151.4 ± 2.4 ^c^	208.3 ± 0.49 ^bc^	262.7 ± 8.59 ^bc^	309.5 ± 38.4 ^b^	169.6 ± 6.86 ^c^	241.4 ± 44.3 ^bc^
Histidine	296.7 ± 2.56 ^b^	297.98 ± 0.62 ^b^	382.4 ± 0.15 ^b^	378.2 ± 7.45 ^b^	407.8 ± 44 ^b^	808.1 ± 34.6 ^a^	507 ± 156 ^ab^
***Bitter/sweet/sulfurous taste FAAs***							
Cysteine	25.8 ± 0.18 ^a^	19.5 ± 0.24 ^ab^	18.6 ± 0.06 ^ab^	19.7 ± 0.24 ^ab^	14.3 ± 4.48 ^ab^	11.5 ± 0.12 ^b^	18.1 ± 3.33 ^ab^
Valine	355.98 ± 2.11 ^bc^	331.9 ± 0.58 ^c^	407.4 ± 2.22 ^ab^	384.97 ± 8.02 ^abc^	416.5 ± 1.7 ^a^	349.5 ± 0.52 ^c^	378.8 ± 24.1 ^abc^
Methionine	200.6 ± 1.57 ^de^	195.08 ± 0.52 ^e^	229.6 ± 1.19 ^ab^	213.4 ± 4.03 ^cd^	224.9 ± 4.1 ^abc^	217.5 ± 1.94 ^bc^	233.6 ± 0.59 ^a^
Lysine	377.3 ± 8.55 ^b^	334.6 ± 5.19 ^b^	401.8 ± 4.9 ^b^	505.3 ± 6.87 ^a^	417.8 ± 29.4 ^a^	342.5 ± 5.26 ^b^	374.9 ± 33.8 ^b^
Arginine	467.7 ± 3.31 ^ab^	432.6 ± 0.26 ^b^	493.6 ± 2.18 ^ab^	521.5 ± 11.8 ^a^	497.2 ± 5.23 ^ab^	460.2 ± 4.35 ^ab^	490.4 ± 32.9 ^ab^
Proline	271.9 ± 16.5 ^a^	225.5 ± 0.86 ^a^	260.36 ± 1.73 ^a^	277.51 ± 7.14 ^a^	333 ± 44.1 ^a^	364.7 ± 16.2 ^a^	317.7 ± 54.8 ^a^
Total FAAs	7338.9	6410.3	7395.3	7253.8	7688.3	7370.4	7562.2

The values are the predicted means ± SEM. ^a–e^ Mean values within the same rows with different superscripts are significantly different among treatments (*p* < 0.05).

## Data Availability

Not applicable.
